# Physiological and Transcriptomic Responses of Chinese Cabbage (*Brassica rapa* L. ssp. *Pekinensis*) to Salt Stress

**DOI:** 10.3390/ijms18091953

**Published:** 2017-09-12

**Authors:** Nianwei Qiu, Qian Liu, Jingjuan Li, Yihui Zhang, Fengde Wang, Jianwei Gao

**Affiliations:** 1 Institute of Vegetables and Flowers, Shandong Academy of Agricultural Sciences, Shandong Key Laboratory of Greenhouse Vegetable Biology, Shandong Branch of National Vegetable Improvement Center, Jinan 250100, China; nianweiqiu@163.com (N.Q.); lijj0620@163.com (J.L.); zyh_0923@163.com (Y.Z.); 2 College of Life Science, Qufu Normal University, Qufu 273165, China; happy329820@163.com

**Keywords:** Chinese cabbage, NaCl stress, physiological, transcriptomic, responses to salt treatment

## Abstract

Salt stress is one of the major abiotic stresses that severely impact plant growth and development. In this study, we investigated the physiological and transcriptomic responses of Chinese cabbage “Qingmaye” to salt stress, a main variety in North China. Our results showed that the growth and photosynthesis of Chinese cabbage were significantly inhibited by salt treatment. However, as a glycophyte, Chinese cabbage could cope with high salinity; it could complete an entire life cycle at 100 mM NaCl. The high salt tolerance of Chinese cabbage was achieved by accumulating osmoprotectants and by maintaining higher activity of antioxidant enzymes. Transcriptomic responses were analyzed using the digital gene expression profiling (DGE) technique after 12 h of treatment by 200 mM NaCl. A total of 1235 differentially expressed genes (DEGs) including 740 up- and 495 down-regulated genes were identified. Functional annotation analyses showed that the DEGs were related to signal transduction, osmolyte synthesis, transcription factors, and antioxidant proteins. Taken together, this study contributes to our understanding of the mechanism of salt tolerance in Chinese cabbage and provides valuable information for further improvement of salt tolerance in Chinese cabbage breeding programs.

## 1. Introduction

Salt stress, one of the major environmental stressors, greatly impacts crop yield and quality. According to statistics from the Food and Agriculture Organization (FAO 2008, available on line: http://www.fao.org/ag/agl/agll/spush/), more than 400 million hectares of land are currently affected by salinity, and this area is expanding [1]. In general, salt stress can lead to a series of adverse effects, including ion homeostasis, ionic toxicity, osmotic stress, membrane alterations, and oxidative damage as well as nutrient imbalance at the whole-plant level due to excess sodium ions [2,3,4]. To survive under these adverse environmental conditions, plants have evolved complex tolerance mechanisms, including the production of antioxidants and compatible osmolytes, reduced sodium absorption, and compartmentalization of toxic sodium ions away from the cytoplasm to reduce molecular damage [2,5].

In past decades, many studies have explored the salt tolerance mechanism at the molecular level in plants. After sensing the salt stress signal, signal transduction pathways are activated and a large number of defense response-related genes are induced, ultimately conferring salt tolerance on the plant. The proteins encoded by these genes that are induced in this response process can be divided into five subgroups: (1) proteins involved in signal transduction, such as MAPK (mitogen-activated protein kinase), SOS (salt overly sensitive), and other proteins; (2) transcription factors (TFs), such as bZIP (basic-leucine zipper domain), MYB (v-myb avian myeloblastosis viral oncogene homolog), ERF (ethylene-responsive factors), WRKY, and NAC; (3) proteins involved in osmolyte synthesis, such as BADH (betaine aldehyde dehydrogenase), CMO (choline monooxygenase), P5CS (Δ-1-pyrroline-5-carboxylate synthase), and SS (sucrose synthase); (4) antioxidant proteins, such as SOD (superoxide dismutase), POD (peroxidase), CAT (catalase), and GST (Glutathione S-transferase); and (5) other genes induced by salt treatments, such as HSP (heat shock protein), P450, ERD (dehydration-induced protein), and LEA (late embryogenesis abundant) protein [6,7].

Chinese cabbage (*Brassica rapa* L. ssp. *Pekinensis*) is a subspecies of *B. rapa* (AA, 2*n* = 2*x* = 20), and is one of the most important vegetables in Asia, especially in China, Korea, and Japan. It has been documented that a high concentration of salt negatively influences seed germination, seedling growth, leafy head development, and fruit formation [8]. At present, there were some reports on molecular breeding for salt tolerance using molecular markers, such as microsatellite (SSR) in *Brassica* species [9]. However, to our knowledge, there have been no systematic studies performed on the physiological and transcriptomic responses to salt stress in Chinese cabbage.

In this study, the fresh weight of the whole plant, the maximal photochemical efficiency of PSII (Fv/Fm), proline content, and other physiological parameters were measured in Chinese cabbage after treatment by NaCl. Additionally, the transcriptomic responses to salt stress were detected using the digital gene expression profiling (DGE) method. The objectives of this study were: (1) to analyze the physiological responses of Chinese cabbage under salt-stress conditions; (2) to determine the molecular mechanism of salt-stress tolerance in Chinese cabbage; and (3) to promote future genetic engineering strategies directed towards enhancing salt stress tolerance in Chinese cabbage.

## 2. Results and Discussion

### 2.1. Physiological Influence of Salt Treatment on Chinese Cabbage

**Figure 1:**
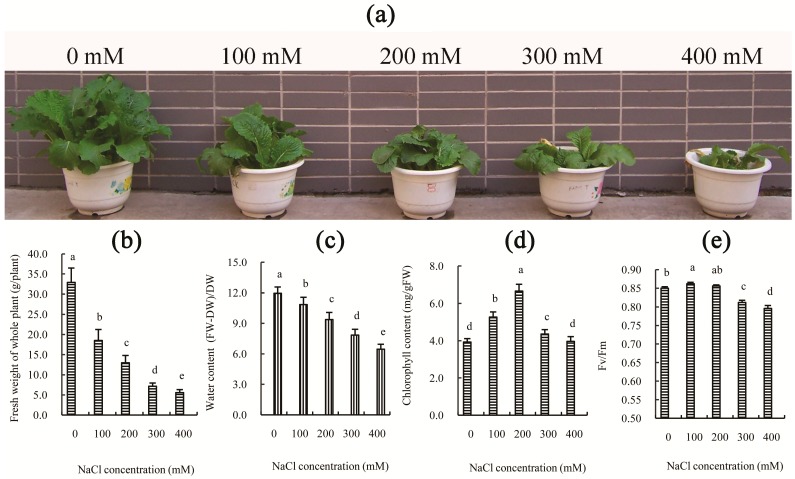
Effects of different concentrations of NaCl on: the growth of whole plants (**a**,**b**); water content (**c**); chlorophyll content (**d**); and photochemical efficiency (Fv/Fm) of PSII (**e**). All parameters were measured on the Day 30 after salt treatment. The values are the means (±SD) of 15 replicates. Means followed by different letters indicate significant differences (*p* < 0.05).

A series of cellular changes occur when undergoing salt stress, including water deficits, ion homeostasis, ionic toxicity, membrane alterations, and free radical production, resulting in slow growth and/or death [2]. Previous studies have shown that treatment with different concentrations of NaCl solutions, such as 100 mM or higher, can significantly inhibit seed germination and seedling growth in Chinese cabbage [8]. In this study, the growth and the water content of Chinese cabbage were also significantly reduced by various concentrations of NaCl solution, and this effect became more pronounced following an increase in NaCl concentration at 30 days after treatment (Figure 1a–c). However, the chlorophyll content was not reduced in the NaCl-treated leaves and even increased significantly in the 200 mM NaCl-treated leaves (Figure 1d), although this may be partly due to inhibition of growth. Additionally, the reduction of growth of Chinese cabbage after 100 and 200 mM NaCl treatment was correlated with the decrease in photosynthetic capacity (Table 1), rather than photosystem impairment (Figure 1e). However, following an additional increase in NaCl concentration (300 or 400 mM NaCl), both the decline of photosynthetic capacity and the impairment of photosystems likely contributed to the inhibition of growth of Chinese cabbage. This conclusion is supported by the photosynthetic indexes (Table 1), where the net photosynthetic rate (Pn), stomatal conductance (Gs), intercellular CO_2_ concentration (Ci), and transpiration rate (Tr) all significantly decreased (*p*-value < 0.05) as the concentration of salt increased. However, the maximal efficiency of PSII photochemistry (Fv/Fm) did not decrease after salt treatment for 30 days at concentrations below 300 mM (Figure 1e). Furthermore, our results indicated that the decrease of P_n_ in the salt-treated leaves is primarily due to the decline of Gs and Ci. These results are consistent with our previous studies on Chinese cabbage cultivar “Juhong 65”, which can survive for long periods of time at 200 mM NaCl, but can only complete an entire life cycle at 0–100 mM NaCl [8]. These results indicated that, as a glygophyte, Chinese cabbage has higher salt tolerance. Thus, based on the above results, 200 mM NaCl was selected as the optimal concentration for further physiological and transcriptomic analyses of Chinese cabbage.

### 2.2. Physiological Responses of Chinese Cabbage to Salt Stress

**Figure 2:**
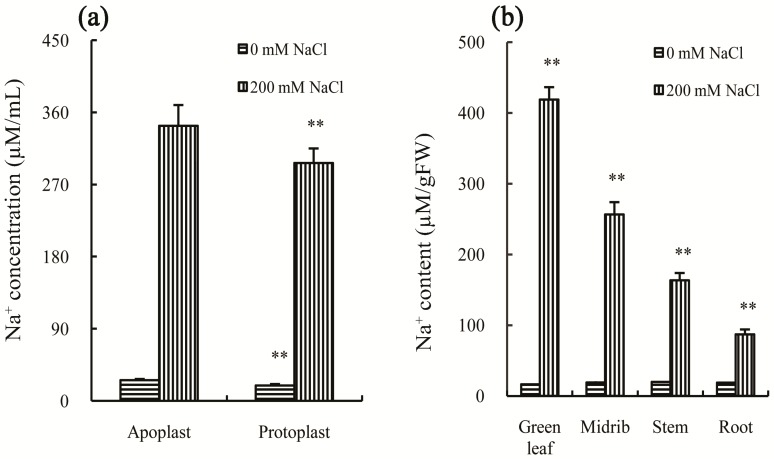
Effects of 200 mM NaCl stress on: the Na^+^ concentration in the apoplast and protoplast (**a**); and the Na^+^ content in green leaves, midribs, stems and roots (**b**). All parameters were measured on the Day 30 after salt treatment. The values are the means (±SD) of five replicates. ** Indicate *p* < 0.01.

To cope with salt stress, plants have evolved many methods to reduce the damage. The common method is to quarantine the cytoplasm away from high concentrations of Na^+^. There are four ways to achieve this goal, including salt excretion, salt dilution, salt accumulation, and salt exclusion [3,10]. However, Chinese cabbage has no salt-avoidance mechanisms with the capacity to reduce the damage caused by salt stress. Firstly, Chinese cabbage has no salt gland to secrete Na^+^ out of the plant. Secondly, it cannot dilute the Na^+^ by the way of more rapid growth or increased absorption of water, since its growth was significantly inhibited by salt stress, and water content decreased after salt treatment (Figure 1a–c). Thirdly, the Na^+^ content in apoplast was significantly higher (*p*-value < 0.05) than that in protoplast after treatment by 200 mM NaCl for 30 days (Figure 2a), suggesting that it cannot (unlike salt-accumulating plants) sequester the Na^+^ in a vacuole as an osmoprotectant. Finally, Chinese cabbage has no capacity to prevent salt absorption or to transport it to the leaves as a salt-exclusion plant, since the leaves were found to have the highest Na^+^ concentration after treatment by 200 mM NaCl for 30 days, followed by the midribs, stems, and roots (Figure 2b). These results also confirm the proposal that Chinese cabbage belongs to the glycophyte class of plants [8].

**Figure 3:**
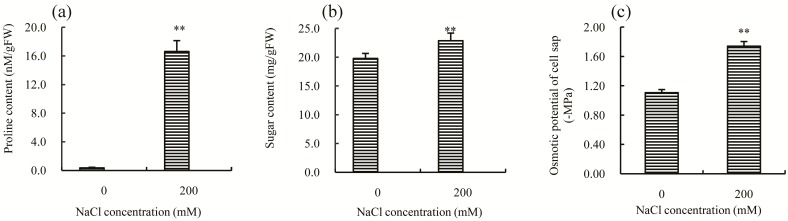
Effects of 200 mM NaCl stress on the: proline (**a**); and soluble sugar (**b**) concentrations; and the osmotic potential (**c**), in the leaves of Chinese cabbage seedlings. All parameters were measured on the Day 30 after salt treatment. The values are the means (±SD) of five replicates. ** Indicate *p* < 0.01.

Other mechanisms that are universally employed by plants to enhance salt tolerance and to cope with salt stress include the production of osmolytes, increasing the capacity of scavenging ROS (reactive oxygen species), and maintaining the balance of Na^+^/K^+^ [2,10]. Proline and soluble sugars are two kinds of effective osmolytes that can reduce osmotic damage caused by salt stress [11]. After 30 days of treatment with 200 mM NaCl, both proline (Figure 3a) and soluble sugars (Figure 3b) significantly increased (*p*-value < 0.05); notably, the concentration of proline was about 47–fold higher than the control, suggesting that this might be the principal reason for the decline observed in the osmotic potential of cell sap (Figure 3c). Thus, modulation of proline concentration significantly contributes to increased salt tolerance in Chinese cabbage.

**Figure 4:**
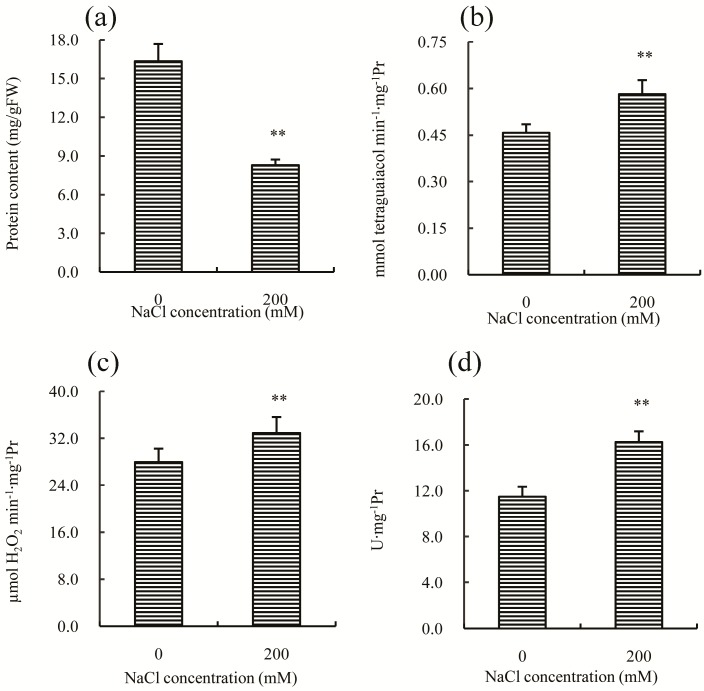
Effects of 200 mM NaCl stress on: the soluble protein content (**a**); and the enzyme activities of: POD (peroxidase) (**b**); CAT (catalase) (**c**); and SOD (superoxide dismutase) (**d**), in the leaves of Chinese cabbage seedlings. All parameters were measured on Day 30 after salt treatment. The values are the means (±SD) of five replicates. ** Indicate *p* < 0.01.

Antioxidant enzymes, such as POD (peroxidase), CAT (catalase), and SOD (superoxide dismutase), play important roles in the adaptation of plants to salt stress. In this study, the concentration of total soluble proteins was first measured, and the result indicated a significant decrease compared to the control (Figure 4a), which may be a common response to salt stress in many plants [12,13,14], due to the ability of sodium and chloride to inhibit protein synthesis [15]. The activity of POD, CAT, and SOD increased significantly after salt treatment (Figure 4b–d), suggesting that salt stress induced the synthesis of proteins in the antioxidant enzyme system, or that any inhibitory mechanisms in Chinese cabbage were inactivated by salt treatment. This result is consistent with previous studies on other plants, such as rice [16], chickpea [17] and alfalfa [18], in which the activities of antioxidant enzymes also increase after salt treatment.

**Figure 5:**
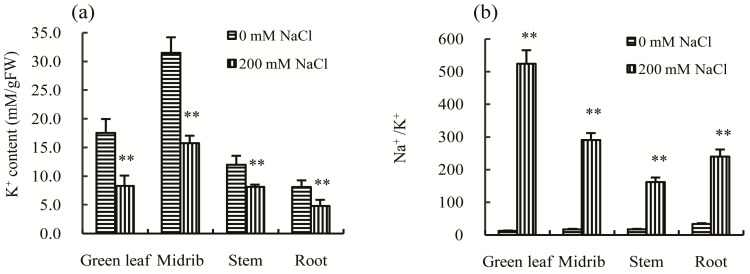
Effects of 200 mM NaCl stress on: the K^+^ concentration (**a**); and the Na^+^/K^+^ ratio (**b**), in the green leaves, midribs, stems, and roots of Chinese cabbage seedlings. All parameters were measured on Day 30 after salt treatment. The values are the means (±SD) of five replicates. ** Indicate *p* < 0.01.

Maintaining a lower Na^+^/K^+^ ratio in the cytoplasm is necessary for cell function in plants. Therefore, the ability to maintain a lower Na^+^/K^+^ ratio in the cytoplasm of plants when under salt stress is critical. For example, the concentration of K^+^ in the halophyte *Suaeda maritime* increases with increased NaCl concentration, maintaining a relatively stable Na^+^/K^+^ ratio in the cytoplasm [19]. However, excessive Na^+^ absorbed into the cytoplasm eventually leads to competitive inhibition of K^+^ uptake, resulting in a decrease in the concentration of K^+^ for most plants under salt stress [20]. In this study, the concentration of K^+^ was found to be significantly lower in green leaves, midribs, stems, and roots (Figure 5a), while the Na^+^/K^+^ ratios in the above organs were all significantly increased after salt treatment (Figure 5b). These results suggest that Chinese cabbage does not have the capacity to maintain a balanced Na^+^/K^+^ ratio to enhance tolerance to salt stress.

**Figure 6:**
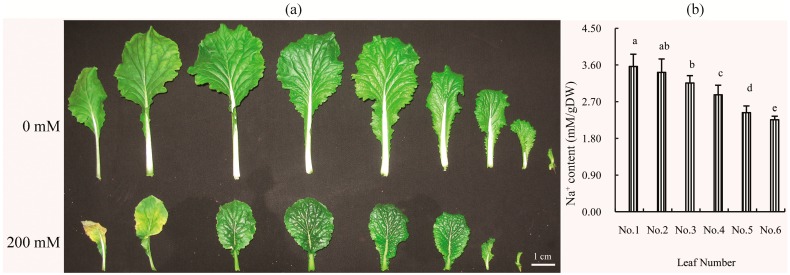
Effects of 200 mM NaCl stress on: the senescence (**a**); and the Na^+^ content (**b**), in different leaf positions of plants exposed to 200 mM NaCl. The Na^+^ concentrations were measured on Day 30 after salt treatment. The values are the means (±SD) of five replicates. Means followed by different letters indicate significant differences (*p* < 0.05). The order of old leaves to young leaves is from left to right. Leaf number from No. 1 to No. 6 is from oldest leaf to younger leaf. The No. 1 leaf is the oldest leaf.

Additionally, it is possible that salinity, as an important environmental stressor, could promote leaf senescence and thus affect crop yield. However, previous studies have shown that nutrients accumulated in senescent leaves can be exported to actively growing organs such as young leaves, thus allowing for fruit development during senescence [21]. In Figure 6a, the old leaves of a Chinese cabbage plant can be seen to be yellow and senesced, while the young leaves are still green after 30 days of treatment by 200 mM NaCl (Figure 6a). Additionally, the senescing leaves accumulated more NaCl than the young leaves (Figure 6b). Therefore, we propose that the course of leaf senescence is a mechanism by which the plant can resist high salinity, since this method would remove excess Na^+^ from the plant by loss of the senesced leaves, allowing the plant as a whole to complete its life cycle. For example, although the Chinese cabbage is significantly inhibited by different concentrations of salt, it can still complete an entire life cycle under 100 mM NaCl treatment [8].

### 2.3. Transcriptomic Responses of Chinese Cabbage to Salt Stress

When suffering from salt stress, first the signal transduction pathways will be activated, and then a series of defense response-related genes will be induced, including those encoding TFs, proteins involved in osmolyte synthesis, antioxidant proteins, and other proteins that can confer salt tolerance on plants [6,7]. To explore the molecular mechanism of salt tolerance in Chinese cabbage, the RNA expression profile of both a salt-treated and a control Chinese cabbage were detected using DGE method. Additionally, to reduce false positive rates, two biological replicates each from salt-treated and control plants were sequenced, and the gene expression correlations between the two biological replicates were analyzed. The Pearson *r* values were 0.987 and 0.995 for control (Figure S1a) and salt treatment (Figure S1b), respectively, suggesting that the data obtained from these two biological replicates were reliable. In this study, based on a threshold of divergent probability ≥0.8 and an absolute value of log2 Ratio ≥1, a total of 1235 significantly differentially expressed genes (DEGs), including 740 up-regulated and 495 down-regulated, were identified after 12 h of treatment by 200 mM NaCl (Table S1). Additionally, to validate the DGE results, an RT-qPCR analysis was performed, and the results showed that the expression patterns of the selected 38 genes were uniform between the DGE and the RT-qPCR databases (Table S2). Furthermore, based on their functions, the up-regulated DEGs that related to the plant defense response were classified into five subgroups, including those homologous to signal transduction proteins, TFs, proteins related to osmolyte synthesis, antioxidant proteins, and other proteins that could confer salt tolerance on plants. Of course, some genes involved in negative regulating salt tolerance were identified from the down-regulated DEGs. Additionally, the down-regulated genes that involved in salt stress response were also analyzed.

### 2.4. Up-Regulated Genes Involved in Signal Transduction

One important role of the SOS pathway is to activate the *SOS1* (salt overly sensitive 1) gene, which encodes a Na^+^/H^+^ antiporter with the function of expelling excess Na^+^ from the cell, thereby maintaining cellular ion homeostasis [22]. *SOS2* encodes a CBL-interacting protein kinase that can interact with SOS3 (calcineurin B-like protein), thus activating SOS1 in the SOS pathway [3]. In this study, five genes homologous to *SOS2* were significantly up-regulated after salt treatment (Table 2), suggesting that they play a specific role in transducing the salt stress signal.

Calcium, a ubiquitous secondary messenger, plays an important role in the cellular response to various environmental stresses [23,24]. In plants, four groups of Ca^2+^ sensors, including calmodulins (CaM), calmodulin-like proteins (CMLs), calcium-dependent protein kinases (CDPKs) and calcineurin B-like proteins (CBLs) are responsible for the recognition of the Ca^2+^ signal [25,26,27]. In this study, two *CMLs* were found to be significantly up-regulated at 12 h after salt treatment (Table 2), suggesting that they could play important roles in the salt stress response in plants. Previous studies also support this theory, as overexpression of the rice *MSR2* gene, which encodes a CML protein, enhances salt tolerance in *Arabidopsis* [28], while knockout of the *AtCML* gene results in a salt-sensitive phenotype [29].

Many studies have shown that the MAPK cascades are significant salt-stress signal transducers [30]. The MAPK cascades are modulated by three classes of protein kinases, including MAPK kinase kinases (MAPKKKs), MAPK kinases (MAPKKs) and MAPKs [31] Previous studies indicate that the *MAPK* genes can be induced by salt treatment in a range of plant species [32,33,34], and overexpression of the *MAPK* genes can increase the salt tolerance of plants [35]. Additionally, as a protein involved in the first steps of the MAPK cascade, MAPKKK can also be induced by salt stress, and plays an important role in increasing the salt tolerance of plants [36]. Based on our data, one *MAPKKK* gene was significantly induced by salt treatment (Table 2), suggesting that MAPKKK is vital to the salt response in Chinese cabbage. However, genes homologous to *MAPK* or *MAPKK* were not significantly up-regulated at 12 h after salt treatment. The functions of the MAPK cascades in salt stress are subjected to further investigation.

### 2.5. Up-Regulated Genes Encoding Transcription Factors

Transcription factors play an important role in response to various biotic or abiotic stresses by regulating specific downstream genes [37,38]. In recent years, many studies have shown that TFs are essential in mediating salt stress response. For example, overexpression of *NAC* [39], *MYB* [40], heat shock transcription factor (*HSF*) [41], *WRKY* [42], and *bHLH* [43] transcription factors can significantly improve salt tolerance in plants. In this study, 39 genes homologous to *NAC*, *MYB*, *HSF*, *WRKY*, *bHLH*, *ERF*, homeo-box (*HB*), and other *TFs*, were significantly up-regulated by salt treatment (Table 3). This result is consistent with previous reports in other plants, including flax [44], *Arabidopsis* [45] and radish [46], where 230, 33 and 52 *TFs* were reported to be significantly up-regulated by salt treatment, respectively. Our results indicate that these *TFs* may play vital roles in salt tolerance in Chinese cabbage. Further investigation of these genes may reveal more information regarding their function in the regulation of the salt-stress response.

### 2.6. Up-Regulated Genes Encoding Proteins Related to Osmolyte Synthesis

Osmotic stress is one of the common adverse effects caused by salt stress [47]. To minimize the damage caused by osmotic stress, plants have evolved a mechanism that involves accumulating a large amount of compatible osmolytes, such as proline and soluble sugars [2,11]. In this study, several genes that are involved in osmolyte synthesis, including three *P5CSs* and one *SS*, were observed to be significantly up-regulated after salt stress (Table 4). This result is consistent with our findings that the content of proline and soluble sugars significantly increased after salt treatment (Figure 3). Additionally, many studies have reported that over-expression of *P5CS* [48,49] increases salt tolerance in transgenic plants. In light of these results, it is likely that the increased expression of the above genes can result in osmolyte accumulation, which may be an important factor in contributing to salt tolerance in Chinese cabbage.

### 2.7. Up-Regulated Genes Encoding Antioxidant Proteins

Salt stress leads to oxidative stress via rapid and excessive production of reactive oxygen species (ROS), such as H_2_O_2_, OH^●^ and O₂^●−^, which can cause oxidative damage [3]. As a survival mechanism, plants have evolved a set of complex antioxidant defense systems, including antioxidant enzymes, such as SOD, POD, CAT, and GST. Many studies have shown that the activity of antioxidant enzymes can be induced by salt stress [50,51], and overexpression of antioxidant enzymes can mitigate the damage caused by salt treatment [52,53,54]. In the current study, genes homologous to POD (five genes), GST (five genes), CAT (three genes), thioredoxin superfamily protein (one gene) and ferritin 1 (one gene) were observed to be significantly up-regulated after salt treatment (Table 5). This result was similar to previous findings in radish, where 34 genes encoding antioxidant enzymes, such as POD, SOD, and GST are significantly up-regulated by salt stress [46]. Therefore, it can be reasonably inferred that antioxidant defense systems play a critical role in the salt stress response in Chinese cabbage.

### 2.8. Other Genes Induced by Salt Treatment

LEA proteins are a group of proteins that accumulate in the last stages of seed development, and play important roles in response to various stresses. For example, overexpression of *LEA* genes can improve drought, salt, and freezing stress tolerance in transgenic plants [55,56]. LEA proteins function to improve enzyme activity and to stabilize membranes under various stress conditions [57,58,59]. In this study, the expression of 19 *LEA* genes, including six dehydrin genes that belong to the second subgroup of *LEA* genes [60], were strongly induced by salt stress (Table 6), suggesting that they may be involved in the salt stress response in Chinese cabbage. This result is consistent with previous findings in radish where five *LEA* genes were significantly up-regulated after salt treatment [46].

Improving the capacity of water absorption is an efficient strategy that can be used to resist osmotic stress. In plants, the transmembrane transport of water is primarily carried out by water channel proteins. Aquaporins, a class of water channel proteins, play an important role in the uptake of groundwater during stress-induced water deficits in plants [61,62]. The transcription level of aquaporin mRNA can be influenced by various stresses, including drought, salt, and low temperature [63]. It has been reported that PIP (plasma membrane intrinsic protein) expression can contribute to water uptake in roots, and promote growth recovery in salinized plants [64]. In the current study, two *PIP* genes were significantly induced by salt treatment (Table 6), suggesting that this protein may promote the transmembrane transport of water during osmotic adjustment.

Leaf senescence is a mechanism that allows Chinese cabbage to complete its life cycle under salt stress. However, the molecular mechanism is still unknown in Chinese cabbage. Previous studies have reported that the senescence-associated gene (*SAG*) is involved in the regulation of leaf senescence [65,66]. In our study, three genes homologous to senescence-associated gene 29 were significantly induced by salt stress. These results are also consistent with the hypothesis that leaf senescence is important for salt tolerance in Chinese cabbage, and suggest that these *SAG* genes may be involved in this regulatory process.

Additionally, several genes that are homologous to HSP, P450, ERD were also significantly up-regulated by salt treatment (Table 6). These results are consistent with previous findings in other plants, including radish [46], barley [67], and cotton [68]. Furthermore, several studies using protein overexpression or mutational analysis have reported that the above genes play essential roles in salt stress response [69,70,71]. Taken together, these results suggest that the mechanism of salt tolerance in Chinese cabbage is very complex and is likely to be modulated by multiple genes.

### 2.9. Down-Regulated Genes Involved in Salt Stress Response

To further understand the mechanisms of salt tolerance in Chinese cabbage, the down-regulated genes were also analyzed, of which more than 40 genes were involved in photosynthesis (Table S1). Additionally, 13 genes that encoding SAUR-like auxin-responsive proteins, seven genes that encoding expansin proteins, three genes that encoding gibberellin-regulated family proteins and other genes that associated with plant growth and development were detected in the down-regulated genes. This result was consisted with the above findings that the photosynthesis and growth were significantly inhibited by salt treatment (Figure 1 and Table 1). Interestingly, some genes, such as peroxidase [54] basic helix-loop-helix (bHLH) [72], bZIP [73] and others that could confer plant salt tolerance were also down-regulated by salt stress (Table S1), suggesting that the transcriptomic response of Chinese cabbage to salt stress is very complex, and this phenomenon is also found in other plants [46,67,68].

## 3. Materials and Methods

### 3.1. Plant Materials and Salt Treatment

The seeds of a Chinese cabbage cultivar (Brand name: Qingmaye, Shandong Weifang Seed Co., LTD, Weifang, China), a variety planted mainly in North China, were germinated in sand moistened with half-strength Hoagland and Arnon’s revised Hoagland solution [74]. When the seedlings had 4–5 leaves, uniform seedlings were treated with full-strength nutrient solution (as a control) or subjected to salt treatment in increments of 100 mM NaCl every 24 h to the final concentration, to avoid salt-shock. Salt treatments consisted of 0, 100, 200, 300, or 400 mM final concentration of NaCl. Final salinity level was achieved at same day. Note that the 0 mM NaCl treatment contained about 2 mM NaCl from the Hoagland solution; NaCl was dissolved in Hoagland nutrient solution and plants were watered daily to capacity with 0.5 L of salt solution. Seedlings were grown in a greenhouse under conditions of 40–50% relative humidity, temperature regimes of 15–25/10–15 °C day/night, photoperiod of 16/8 h light/dark, and maximal illumination of approximately 600 μmol/m^2^·s^1^. For physiological parameter measurements, the seedlings were determined or harvested on Day 30 after the final NaCl concentration was reached.

For transcriptomic expression analysis, uniformly-sized Chinese cabbage seedlings with four fully opened leaves were treated with 200 mM NaCl directly. Whole plants were harvested after 12 h of salt treatment and immediately frozen in liquid nitrogen and stored at −80 °C for subsequent RNA isolation.

### 3.2. Determination of Fresh Weight, Water Content, and Chlorophyll Content

The fresh weight (FW) of the whole plant was recorded on the Day 30 after reaching the final NaCl concentration.

To measure the water content (WC), all the green leaves were separated, and FW were recorded. Green leaf samples were then dried in an oven at 75 °C for 72 h and dry weights (DW) were measured. WC was calculated as: (FW-DW)/DW.

The chlorophyll content of the maximum function leaf per plant was determined according to the method developed by Porra et al. [75]. Chlorophyll was extracted with 80% acetone.

### 3.3. Determination of Na^+^ and K^+^ Concentrations

Dry samples (100 mg) of all green leaves, midribs, stems and roots of plant were ashed at 500 °C in a muffle furnace according to Qiu et al. [76]. The ash was dissolved in concentrated nitric acid and diluted with distilled water; the Na^+^ and K^+^ concentrations were measured using a M410 Flame photometer (Sherwood, UK). The Na^+^ and K^+^ concentrations in Chinese cabbage organs were expressed as mmol/gDW.

### 3.4. Determination of the Maximal Efficiency of PSII Photochemistry (Fv/Fm) and the Photosynthetic Gas Exchange Indexes

Fv/Fm in the dark-adapted state was determined by measuring the modulated light at a continuous light intensity of 3000 μmol·m^−2^·s^−1^ using a Handy PEA (Plant Efficiency Analyser; Hansatech Instrument Ltd., King’s Lyn, UK). Gas exchange analysis was carried out using a Ciras-2 portable photosynthetic system (Hansatech, Hitchin, UK). Leaf net photosynthetic rate (Pn), stomatal conductance (Gs), internal CO_2_ concentration (Ci) and transpiration rate (Tr) were determined at a CO_2_ concentration of 400 μmol/mol, 40% relative humidity, and a saturation light intensity of 1000 μmol·m^−2^·s^−1^. Photosynthetic gas exchange indexes were all determined with the maximum function leaf per plant outdoor at 9:00~11:00 am.

### 3.5. Determination of Proline and Soluble Sugars Content and Osmotic Potential (Ψ_s_)

Proline accumulation in leaves was determined as described by Bates et al. [77]. A total of 1.0 g fresh samples were homogenized in 10 mL 3% sulfosalicylic acid, and the absorbance at 520 nm was recorded using l-proline as a standard.

The soluble sugar content of fresh leaves was determined according to the method developed by Jermyn et al. [78]. Glucose was used to prepare the standard curve of soluble sugars.

The *i*C value of the cell sap squeezed from the leaf tissues was measured using a Vapor Pressure Osmometer (VAPRO 5520; Hansatech Instrument Ltd., King’s Lyn, UK). The tissue osmotic potential of solutes was calculated as Ψ_s_ = −*i*CRT. All leaves were used for determination of proline and soluble sugars content and osmotic potential.

### 3.6. Determination of Soluble Protein Content and Antioxidant Enzyme Assays

All fresh cabbage leaves (1.0 g) were ground with 10 mL cold buffer containing potassium phosphate (50 mM, pH 7.0), 20% (*v*/*v*) glycerol, 1 mM DTT, 1 mM EDTA, 1% PVP (*w*/*v*), and 5 mM MgSO_4_. Leaf extract was initially centrifuged at 12,000 *g* for 6 min, and then centrifuged again at 26,000 *g* for 15 min. The supernatant was separated into EP tubes and stored at −80 °C for subsequent analyses for catalase (CAT, EC 1.11.1.6), peroxidase (POD, EC 1.11.1.7), and superoxide dismutase (SOD, EC 1.15.1.1). The soluble protein concentration in leaf was determined according to the method of Bradford [79], with bovine serum albumin as a protein concentration standard.

The CAT activity was measured by monitoring the absorbance of H_2_O_2_ at 240 nm (extinction coefficient = 27.78 mM^−1^∙cm^−1^) as described by Knöraer et al. [80]. The reaction mixture contained potassium phosphate (50 mM, pH 7.0), 10 mM H_2_O_2_, and 0.1 mL leaf extract in a final volume of 3 mL at 25 °C.

The POD activity was measured by monitoring the absorbance of tetraguaiacol at 470 nm (extinction coefficient = 26.6 mM^−1^∙cm^−1^) as described by Chance and Maehly [81]. The reaction mixture contained potassium phosphate (50 mM, pH 7.0), 5 mM H_2_O_2_, 18 mM guaiacol and 0.02 mL leaf extract in a final volume of 3 mL at 25 °C.

The SOD activity was determined by the method of Beyer and Fridovich [82]. The reaction mixture contained potassium phosphate (50 mM, pH 7.5), 13 mM Met, 75 μM NBT, 10 μM EDTA, 2 μM riboflavin and 0.02 mL leaf extract in a final volume of 3 mL at 25 °C. One unit of SOD was defined as the amount of extract needed for a 50% decrease in the SOD-inhibitable nitro blue tetrazolium (NBT) reduction.

### 3.7. Identification of Differentially Expressed Genes Using DGE

For DGE, total RNA was isolated from 0 mM and 200 mM NaCl-treated Chinese cabbage leaves using a TRNzol-A^+^ reagent according to the manufacturer’s recommendations (Tiangen, Beijing, China). The mRNA was purified from total RNA using Sera-mag Magnetic Oligo (dT) Beads (Illumina Inc., San Diego, CA, USA), and were then fragmented into small strands by adding Illumina fragmentation buffer. The mRNA fragments were then used as templates to synthesize double-stranded cDNAs using a random hexamer primer and the SuperScript Double-Stranded cDNA Synthesis Kit (Invitrogen, San Diego, CA, USA). Thereafter, the purified double-stranded cDNA was ligated to sequencing adapters. Finally, the ligated fragments were enriched by PCR for 18 cycles. Libraries were sequenced using an Illumina HiSeq^TM^ 4000 platform.

To identify differentially expressed genes (DEGs), the adapters, low quality reads (more than half of the bases with a quality score of less than 5), and the reads containing more than 10% unknown bases from raw reads were removed, and the clean reads were aligned to the *B. rapa* (Chiifu-401) reference genome (Available online: http://brassicadb.org/brad/) using the BWA software [83]. After filtering the reads that mapped to multiple reference genes, the unambiguous clean reads were mapped to the reference genes (Available online: http://brassicadb.org/brad/) using the Bowtie software [84]. The total reads that were fully mapped to exons were counted, and the expression levels for each gene were calculated by the RSEM tool [85]. The DEGs were then screened using a Noiseq protocol developed by Tarazona et al. [86]. A threshold, combining divergence probability ≥0.8 and an absolute value of log_2_ Ratio ≥1, was used for the identification of DEGs.

### 3.8. Real-Time Quantitative PCR Validation of the DEGs

For (real-time quantitative PCR (RT-qPCR), total RNA was first isolated from 0 mM and 200 mM NaCl-treated Chinese cabbage seedlings as described in the previous section. The first-strand cDNA was then synthesized using a PrimeScript RT reagent kit with gDNA eraser according to the manufacturer’s recommendations (Takara, Dalian, China). RT-qPCR was performed using a SYBR Green PCR master mix (Takara) on an IQ5 real-time PCR system (Bio-Rad, Hercules, CA, USA). The RT-qPCR primers designed for the selected 38 genes are listed in Table S3. The *actin* gene was used as a constitutive expression control in these experiments. The PCR-cycling conditions comprised an initial polymerase activation step at 95 °C for 1 min, followed by 40 cycles at 95 °C for 10 s, and 60 °C for 30 s. After each PCR run, a dissociation curve was designed to confirm the specificity of the product and to avoid the production of primer dimers. Three replicates of each sample were performed to calculate the average *C*_t_ values. The relative expression level was calculated by the comparative 2^−ΔΔ*C*t^ method [87].

### 3.9. Statistical and Geostatistical Analyses

Data in all figures, Table 1 and Table S2 are represented as mean ±SD. Statistical analyses were performed using the SPSS 17.0 software package. Multiple sets of data were analyzed using One-way factor ANOVA (analysis of variance) model and Duncan’s multiple range test. Means followed by different letters indicate significant differences (*p* < 0.05). Two sets of data were analyzed using Student’s *t*-tests; “*” and “**” indicate *p* < 0.05 and *p* < 0.01, respectively.

## 4. Conclusions

**Figure 7:**
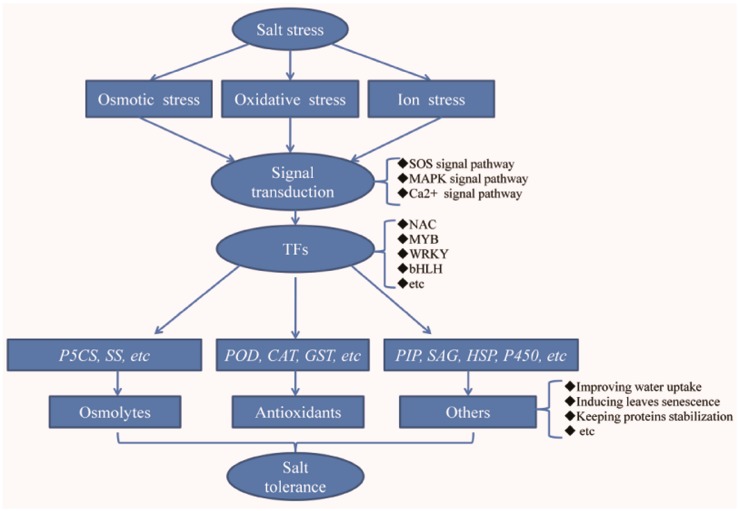
A proposed model of the salt-stress response in Chinese cabbage.

In this study, the influence of salt stress on Chinese cabbage, and the defense response at the physiological and transcriptomic levels in Chinese cabbage were systematically analyzed. Our proposed model for salt tolerance is summarized in Figure 7 and can be briefly described as follows. Firstly, the Chinese cabbage belongs to a glycophyte class with high salt tolerance. Secondly, the primary factors that contribute to salt tolerance are accumulation of osmoprotectants and high activity of enzymes in the antioxidant defense system. Thirdly, the accumulation of Na^+^ in old leaves and subsequent promotion of old leaf senescence help to complete the life cycle under salt stress. Finally, genes involved in signal transduction and osmolyte synthesis, and genes encoding TFs and antioxidant proteins, play critical roles in salt tolerance in Chinese cabbage. The large number of up-regulated genes by salt stress suggests that the salt defense response mechanism in Chinese cabbage is very complex, with contributions from multiple pathways. This study provides a foundation on which to build an understanding of the mechanism of salt tolerance in Chinese cabbage, and provides a basis for developing an engineering strategy towards enhancing salt stress tolerance in Chinese cabbage.

## Figures and Tables

**Table 1:** Effect of salt-treatment on photosynthetic indexes of Chinese cabbage. The values are the means (±SD) of 10 replicates.

NaCl Concentration	*P_n_*/µmol (CO_2_) m^−2^∙s^−1^	*Gs*/mmol (H_2_O) m^−2^∙s^−1^	*Ci*/µmol∙mol^−1^	*Tr*/mmol (H_2_O) m^−2^∙s^−1^
0 mM	15.72 ± 1.02 a	141.7 ± 19.0 a	193.8 ± 17.9 a	2.30 ± 0.14 a
100 mM	11.92 ± 0.89 b	84.7 ± 8.5 b	151.7 ± 13.1 b	1.48 ± 0.12 b
200 mM	9.58 ± 0.87 c	58.0 ± 5.6 c	114.5 ± 14.8 c	1.17 ± 0.11 c
300 mM	5.20 ± 1.23 d	26.2 ± 6.4 d	88.2 ± 12.7 d	0.57 ± 0.10 d
400 mM	2.13 ± 0.34 e	10.5 ± 3.8 e	67.8 ± 7.6 e	0.26 ± 0.07 e

*P_n_*, Leaf net photosynthetic rate; *Gs*, stomatal conductance; *Ci*, internal CO_2_ concentration; *Tr*, transpiration rate. Means followed by different letters indicate significant differences (*p* < 0.05).

**Table 2:** The up-regulated differentially expressed genes (DEGs) that may be involved in signaling transduction in Chinese cabbage.

Gene ID	log_2_ Ratio (Treatment/CK)	Probability	Annotation (BlastX to *A. thaliana*)
Bra015388	5.58	0.90	mitogen-activated protein kinase kinase kinase 18
Bra009003	2.95	0.87	CBL-interacting protein kinase 5
Bra023777	1.27	0.81	CBL-interacting protein kinase 7
Bra011236	1.97	0.87	CBL-interacting protein kinase 6
Bra022794	1.94	0.83	CBL-interacting protein kinase 11
Bra010263	1.65	0.84	CBL-interacting protein kinase 6
Bra016962	1.74	0.83	Protein kinase superfamily protein
Bra014542	2.52	0.84	Protein kinase superfamily protein
Bra028679	2.36	0.86	Diacylglycerol kinase1
Bra011033	1.87	0.80	Leucine-rich receptor-like protein kinase family protein
Bra005168	1.69	0.83	Receptor lectin kinase
Bra028545	1.64	0.84	Pyruvate kinase family protein
Bra006864	1.63	0.83	Phosphofructokinase 7
Bra022870	1.36	0.80	Leucine-rich repeat protein kinase family protein
Bra033745	3.16	0.81	Calmodulin like 43
Bra012889	2.79	0.88	Calmodulin-like 41
Bra025654	2.21	0.86	Calcium-binding EF-hand family protein
Bra017927	1.58	0.82	Calcium-binding EF-hand family protein
Bra000430	1.56	0.84	Calcium-binding EF-hand family protein
Bra016936	1.54	0.84	Calcium-binding EF-hand family protein
Bra004620	1.51	0.84	Calcium-binding EF-hand family protein
Bra039661	1.51	0.84	Sodium/calcium exchanger family protein/calcium-binding EF hand
Bra028148	1.38	0.81	Calcium-dependent lipid-binding (CaLB domain) family protein

**Table 3:** The up-regulated DEGs encoding transcription factors in Chinese cabbage.

Gene ID	log_2_ Ratio (Treatment/CK)	Probability	Annotation (BlastX to *A. thaliana*)
Bra007637	4.10	0.89	Homeobox 12
Bra014417	1.83	0.85	Homeobox 12
Bra039116	1.44	0.83	Homeobox 1
Bra039265	3.75	0.90	Homeobox 7
Bra025658	3.47	0.83	NAC domain containing protein 6
Bra019052	3.38	0.89	NAC (No Apical Meristem) domain transcriptional regulator superfamily protein
Bra026353	3.11	0.88	NAC (No Apical Meristem) domain transcriptional regulator superfamily protein
Bra004385	2.90	0.86	NAC-like, activated by AP3/PI
Bra018998	2.28	0.87	NAC domain containing protein 19
Bra008849	1.70	0.80	NAC domain containing protein 83
Bra006186	1.44	0.81	NAC domain containing protein 83
Bra016441	3.12	0.85	PLATZ transcription factor family protein
Bra017670	3.10	0.86	GATA transcription factor 3
Bra040092	3.09	0.87	Integrase-type DNA-binding superfamily protein
Bra020017	3.06	0.84	Phytochrome interacting factor 3
Bra001806	2.93	0.83	Nuclear factor Y, subunit A9
Bra010049	2.85	0.86	Heat shock transcription factor B2A
Bra013253	4.19	0.88	Heat shock transcription factor C1
Bra039022	2.52	0.87	CCCH-type zinc finger family protein
Bra011087	2.08	0.87	Zinc finger C-x8-C-x5-C-x3-H type family protein
Bra003500	1.81	0.82	Basic region/leucine zipper motif 53
Bra001752	1.92	0.84	Zinc-finger protein 2
Bra009464	2.18	0.80	Zinc finger of Arabidopsis thaliana 6
Bra011485	2.47	0.88	Abscisic acid responsive elements-binding factor 3
Bra019645	1.88	0.86	A20/AN1-like zinc finger family protein
Bra040260	2.12	0.85	Abscisic acid responsive elements-binding factor 2
Bra011545	2.27	0.87	G-box binding factor 6
Bra004550	2.10	0.84	G-box binding factor 3
Bra017664	1.56	0.81	G-box binding factor 6
Bra012337	2.27	0.85	Myb domain protein 3
Bra024526	1.99	0.82	Myb domain protein 3
Bra028707	2.19	0.82	WRKY DNA-binding protein 26
Bra010231	1.81	0.82	WRKY DNA-binding protein 11
Bra039409	1.79	0.84	AtBS1(activation-tagged BRI1 suppressor 1)-interacting factor 1
Bra036061	1.66	0.84	cooperatively regulated by ethylene and jasmonate 1
Bra039658	1.66	0.82	Ethylene response factor 8
Bra021200	1.33	0.81	Ethylene-responsive element binding protein
Bra011700	1.52	0.82	GRAS family transcription factor
Bra018896	2.43	0.87	Basic helix-loop-helix (bHLH) DNA-binding superfamily protein

**Table 4:** The up-regulated DEGs encoding proteins related to osmolyte synthesis in Chinese cabbage.

Gene ID	log_2_ Ratio (Treatment/CK)	Probability	Annotation (BlastX to *A. thaliana*)
Bra017051	5.19	0.86	Δ^1^-pyrroline-5-carboxylate synthase 1
Bra005012	4.04	0.92	Δ^1^-pyrroline-5-carboxylate synthase 1
Bra007179	2.34	0.87	Δ^1^-pyrroline-5-carboxylate synthase 2
Bra036282	3.24	0.84	Sucrose synthase 3

**Table 5:** The up-regulated DEGs encoding antioxidant proteins in Chinese cabbage.

Gene ID	log_2_ Ratio (Treatment/CK)	Probability	Annotation (BlastX to *A. thaliana*)
Bra016127	1.43	0.82	Peroxidase superfamily protein
Bra013576	1.23	0.81	Peroxidase superfamily protein
Bra009105	3.56	0.83	Peroxidase superfamily protein
Bra039816	2.59	0.88	Peroxidase superfamily protein
Bra029933	4.02	0.91	peroxidase CB
Bra012239	1.69	0.81	Catalase 1
Bra025995	1.74	0.82	Glutathione *S*-transferase TAU 24
Bra018543	1.74	0.85	Glutathione *S*-transferase F3
Bra000875	1.50	0.81	Glutathione *S*-transferase F3
Bra008915	1.65	0.84	Glutathione *S*-transferase
Bra024820	1.33	0.81	Glutathione *S*-transferase zeta 1
Bra005677	2.68	0.89	Ferretin 1
Bra011408	1.49	0.83	Thioredoxin superfamily protein
Bra007718	1.48	0.81	Thioredoxin superfamily protein
Bra004455	1.45	0.82	Thioredoxin family protein

**Table 6:** Other up-regulated DEGs that are proposed to play important roles in the salt-stress response in Chinese cabbage.

Gene ID	log_2_ Ratio (Treatment/CK)	Probability	Annotation (BlastX to *A. thaliana*)
Bra008242	1.61	0.84	Dehydrin family protein
Bra012230	2.80	0.89	Dehydrin family protein
Bra015779	2.36	0.88	Dehydrin family protein
Bra025819	1.97	0.87	Dehydrin family protein
Bra037177	6.82	0.99	Dehydrin family protein
Bra031809	6.89	0.98	Dehydrin family protein
Bra027219	12.22	0.98	Late embryogenesis abundant (LEA) protein
Bra001603	10.30	0.91	Late embryogenesis abundant (LEA) protein
Bra021457	13.63	0.99	Late embryogenesis abundant (LEA) protein
Bra021436	6.75	0.97	Late embryogenesis abundant (LEA) protein
Bra022221	8.59	0.99	Late embryogenesis abundant (LEA) protein
Bra039946	2.11	0.87	Late embryogenesis abundant (LEA) protein
Bra005353	9.83	0.88	Late embryogenesis abundant (LEA) protein
Bra039956	6.66	0.93	Late embryogenesis abundant (LEA) protein
Bra007054	2.77	0.87	Late embryogenesis abundant (LEA) protein
Bra003039	1.44	0.80	Late embryogenesis abundant (LEA) protein
Bra009225	8.91	0.99	Late embryogenesis abundant (LEA) protein
Bra005911	5.92	0.97	Late embryogenesis abundant (LEA) protein
Bra030494	2.79	0.88	Late embryogenesis abundant (LEA) protein
Bra029121	10.09	0.90	Low temperature induced 65
Bra022584	4.93	0.94	Low temperature induced 65
Bra022585	2.64	0.88	Low temperature induced65
Bra002594	5.21	0.94	Highly ABA-induced PP2C gene 1
Bra015579	4.80	0.91	Highly ABA-induced PP2C gene 2
Bra031574	3.73	0.83	Highly ABA-induced PP2C gene 2
Bra040610	3.70	0.90	AIG2-like (avirulence induced gene) family protein
Bra025365	1.68	0.82	AIG2-like (avirulence induced gene) family protein
Bra021385	1.33	0.81	AIG2-like (avirulence induced gene) family protein
Bra025649	1.50	0.83	AIG2-like (avirulence induced gene) family protein
Bra008661	2.85	0.89	Stress-responsive protein (KIN2)
Bra009620	2.08	0.83	SALT induced serine rich (SIS).
Bra000227	1.44	0.81	Dehydration-induced protein (ERD15)
Bra002217	1.41	0.83	Aluminium induced protein with YGL and LRDR motifs
Bra002216	1.30	0.82	Aluminium induced protein with YGL and LRDR motifs
Bra016644	2.45	0.85	Heat shock protein 70B
Bra015922	2.20	0.83	Heat shock protein 101
Bra013774	1.27	0.81	Heat shock protein 90-7
Bra001078	3.58	0.90	Cytochrome P450, family 87, subfamily A, polypeptide 9
Bra017819	1.85	0.85	Cytochrome P450, family 81, subfamily D, polypeptide 8
Bra023394	7.62	0.98	Senescence-associated gene 29
Bra008850	6.01	0.97	Senescence-associated gene 29
Bra006185	6.01	0.93	Senescence-associated gene 29
Bra000111	1.60	0.82	Plasma membrane intrinsic protein 2E
Bra007100	2.50	0.85	Plasma membrane intrinsic protein 2;5

## Supplementary Materials

Supplementary materials can be found at www.mdpi.com/1422-0067/18/9/1953/s1.
