# Supplementary material for: Physiological and Transcriptomic Responses of Chinese Cabbage (Brassica rapa L. ssp. Pekinensis) to Salt Stress
Source: Int J Mol Sci. 2017 Sep 12;18(9):1953. doi: 10.3390/ijms18091953 (PMC5618602; doi:10.3390/ijms18091953)

Fig. S1 Analysis the gene expression correlations between the two biological replicates in 0 mM (A) and 200 mM (B) NaCl solution treated Chinese cabbage seedlings.

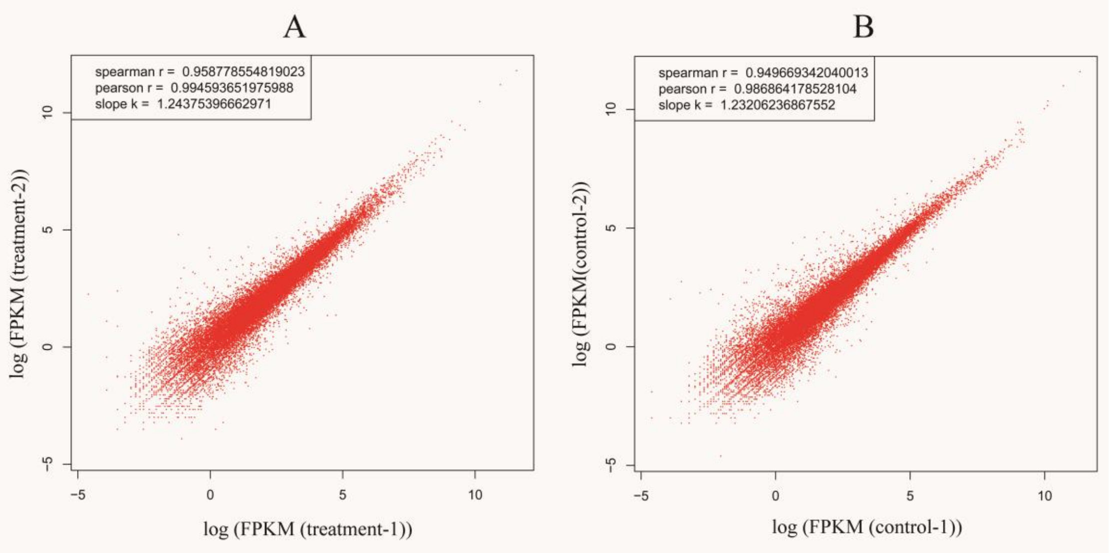

Supplement: Supplementary file 1 [file ijms-18-01953-s001.zip › Fig. S1.pdf]
