# Supplementary material for: Physiological and Transcriptomic Responses of Chinese Cabbage (Brassica rapa L. ssp. Pekinensis) to Salt Stress
Source: Int J Mol Sci. 2017 Sep 12;18(9):1953. doi: 10.3390/ijms18091953 (PMC5618602; doi:10.3390/ijms18091953)
Supplement: Supplementary file 1 [file ijms-18-01953-s001.zip › Table S2.pdf]

Table S2 RNA-Seq experiment validation by RT-qPCR.

Gene ID	RT-q-PCR		RNA-Seq	
	0 mM	200 mM	log_2Ratio(NaCl-200 mM/NaCl-0 mM)	Probability
Bra007637	1	22.24±2.38	4.10	0.89
Bra025658	1	15.95±2.72	3.47	0.83
Bra028707	1	3.84±0.80	2.19	0.82
Bra018896	1	1.36±0.46	2.43	0.87
Bra029121	1	3.35±0.52	10.09	0.90
Bra023394	1	281.25±30.07	7.62	0.98
Bra002594	1	17.80±0.35	5.21	0.94
Bra015388	1	24.09±4.94	5.58	0.90
Bra009003	1	6.64±0.99	2.95	0.87
Bra033745	1	20.23±3.50	3.16	0.81
Bra009105	1	17.34±4.53	3.56	0.83
Bra017051	1	24.91±1.66	5.19	0.86
Bra027219	1	32.80±3.51	12.22	0.98
Bra036282	1	10.28±1.28	3.24	0.84
Bra023777	1	1.29±0.01	1.27	0.81
Bra013774	1	2.45±0.23	1.27	0.81
Bra018469	1	3.82±0.60	1.28	0.81
Bra030498	1	2.14±0.16	1.28	0.81
Bra013911	1	2.47±0.37	1.28	0.80
Bra007683	1	4.37±1.00	1.28	0.81
Bra001886	1	1.99±0.48	1.29	0.81
Bra000315	1	1.54±0.19	1.29	0.80
Bra002216	1	2.82±0.14	1.30	0.82
Bra029697	1	2.86±0.37	1.30	0.80
Bra037761	1	0.30±0.10	-1.20	0.80
Bra010717	1	0.36±0.10	-1.20	0.80
Bra014473	1	0.31±0.05	-1.22	0.80
Bra025297	1	0.30±0.03	-1.22	0.80
Bra027083	1	0.14±0.01	-1.23	0.81
Bra033901	1	0.26±0.05	-1.23	0.80
Bra023597	1	0.24±0.02	-1.24	0.80
Bra028901	1	0.37±0.04	-1.24	0.81
Bra029188	1	0.26±0.03	-1.25	0.81
Bra038805	1	0.34±0.07	-1.25	0.81
Bra032734	1	0.50±0.08	-1.26	0.81
Bra035054	1	0.36±0.01	-1.26	0.80
Bra037953	1	0.46±0.11	-1.26	0.81
Bra027021	1	0.42±0.09	-1.26	0.81
