# Supplementary material for: Physiological and Transcriptomic Responses of Chinese Cabbage (Brassica rapa L. ssp. Pekinensis) to Salt Stress
Source: Int J Mol Sci. 2017 Sep 12;18(9):1953. doi: 10.3390/ijms18091953 (PMC5618602; doi:10.3390/ijms18091953)
Supplement: Supplementary file 1 [file ijms-18-01953-s001.zip › Table S3.pdf]

Table S3 Primers used for RT-qPCR.

Gene ID	Primers (5’-3’)
Bra007637	Forward: AACATTCTCAGAGCCAACTACAA
	Reverse: GAGCAATCCCTTGATTTATCCTAA
Bra025658	Forward: ATTCCTTCCCTGGTTCGTCTA
	Reverse: GATTCTCCCATACCGGCTAAA
Bra028707	Forward: ATGGGAAGGAGGAGCACTTTGAT
	Reverse: CTTTGTGAGGCAGTTTGGATACG
Bra018896	Forward: CCACCGACACGAGCAACAAC
	Reverse: AGGAGCCATCTCACCGACCT
Bra029121	Forward: TCAGCGACTTCGTATGTAACCG
	Reverse: GGAGTCAACTTATCCGCCACAG
Bra023394	Forward: TCACGCTTTCTTTCTTCCTCAC
	Reverse: CGACAAACCTAACGGACTCATC
Bra002594	Forward: TATCACTTCTGCGGCGTTTA
	Reverse: ATCAGCCTCAAGCTCCTCCC
Bra015388	Forward: GACAGCGACGCAATTATTAAACC
	Reverse: TCCTCTTCCACTGACCTCCAGAA
Bra009003	Forward: AAGGTTCAGTCGGTGTTCACG
	Reverse: CCTTCGCCGTCTTCACATTCA
Bra033745	Forward: CGCAGCACTCCACAGAACACTA
	Reverse: CACCGTCTTGGTCAAACACTTT
Bra009105	Forward: ACCCAAACCGCAATTCTGC
	Reverse: GCTTGACTCGCCGTTCTCG
Bra017051	Forward: AGAAGCAAGGTCGTTCAAGCA
	Reverse: CCTCAGCACCAAGTCCAAATC
Bra027219	Forward: TGAGCCAGACAGGTGAGCAAGT
	Reverse: CAGTGGTCCGAGTAGTACCAGTAG
Bra036282	Forward: GTTGCATCGTTAATGGCACATA
	Reverse: CTTTCGTATTGACCGACCGTGT
Bra023777	Forward: GTTCACGGCGAAGGTTTCAG
	Reverse: CAACCACCACAACACCCACA
Bra013774	Forward: ACCGTTTGGCTGATACTCCC
	Reverse: TCGATCAAAGCCGTCTGGTA
Bra018469	Forward: ACCTCAAGAGGCAGATGACTGA
	Reverse: AGCAGAATTGACAACGTGGAAT
Bra030498	Forward: GGTCAAGGCTGTAATGGTGGC
	Reverse: GGTGTAGGGTAAGCGGGTTCA
Bra013911	Forward: GGCGAAGATTCTGATGATGGTT
	Reverse: ATGGATGGATTGTATGGCACAG
Bra007683	Forward: CAATAACACCCTAATCCTCCTTT
	Reverse: TATAACTTCCTTGGCATCTCCTC
Bra001886	Forward: CAATGGTTGGATTCGTGGCT

	Reverse: TGGACTTGGACTCGGCTGTT
Bra000315	Forward: CTCAATCTTTCCCAGCCTATCAG
	Reverse: CTAGCTTGGCAGTACCTTCCTCT
Bra002216	Forward: TGGCATTTGCTGATGATATTGAGT
	Reverse: GGCAGTGATCTTGTTCTTAGGGTT
Bra029697	Forward:AATACTCACCTCCTGACTTCTACAC
	Reverse: GTTAATGGGAACTCAAACACCTC
Bra037761	Forward: TGTATCTGCTTCCGCTAATGCT
	Reverse: CGAACTGGACAGTAAACGCTCT
Bra010717	Forward: CCTGAAGACGAGCATGGTGA
	Reverse: AAGGCTGGAGGGATTCTGTT
Bra014473	Forward: ATCTTGATTGGGACTGGCTCTG
	Reverse: CCTTGTACTCATCCGTCTGCTC
Bra025297	Forward: AACATTCGCAAAGAACCGTGAG
	Reverse: CTGAGAACCTGCCTTGAACCA
Bra027083	Forward: ATCAACGGACGGTTCGCTAT
	Reverse: ACCAAGGAAGGGCAGTGTCT
Bra033901	Forward: AAAGGCTGATGATGTTCCTCTAA
	Reverse: AGACGGGAATATCGAAACTACTG
Bra023597	Forward: ACTCCACTCCTCGTCTCCTCT
	Reverse: CTGCTGTTCTCGCTTTGACTT
Bra028901	Forward: CCGTCTTCCCGTCGTTCAGT
	Reverse: CCCACAGCTCACCTCCACAA
Bra029188	Forward: TGATTCCTAATGCCTCTGTTCTC
	Reverse: GCTTCATCCTCCACTATACTCCA
Bra038805	Forward: ATGTGACAGGGAAAGTGACCAAG
	Reverse: ACTGAATAACTCCAGCACCAACG
Bra032734	Forward: TTTCGCCTCCAAGTTCACTA
	Reverse: TCACTTCTGCCTGAGCTTTC
Bra035054	Forward: AGAACCTTGCCTCTTACACTCC
	Reverse: GATCAAATGTTTCAACCGTCAT
Bra037953	Forward: GTTATGGCACGAGACCTGATT
	Reverse: TTGGCAACGGTAGACTGAAGA
Bra027021	Forward: ACGCACAAGAAGCAATCAGG
	Reverse: ACAAGCACGGACATCAACCA
Actin	Forward: GCTTACGTCGCTCTTGACTACG
	Reverse:GATGGTGATGACTTGTCCATCAG
